# Influenza D in Italy: towards a better understanding of an emerging viral infection in swine

**DOI:** 10.1038/s41598-017-12012-3

**Published:** 2017-09-15

**Authors:** Emanuela Foni, Chiara Chiapponi, Laura Baioni, Irene Zanni, Marianna Merenda, Carlo Rosignoli, Constantinos S. Kyriakis, Mario Vittorio Luini, Maria Lucia Mandola, Luca Bolzoni, Arrigo Daniele Nigrelli, Silvia Faccini

**Affiliations:** 1 OIE Reference Laboratory for Swine Influenza, Parma, 43123 Italy; 2 Istituto Zooprofilattico Sperimentale della Lombardia ed Emilia Romagna, Brescia, 25124 Italy; 3 Istituto Zooprofilattico Sperimentale of Piemonte, Liguria and Valle d’Aosta, Turin, 10154 Italy; 4 Center for Vaccines and Immunology College of Veterinary Medicine, University of Georgia, Athens, GA 30602 USA

## Abstract

Influenza D virus (IDV), a new member of the *Orthomyxoviridae* family, was first reported in 2011 in swine in Oklahoma, and consequently found in cattle across North America and Eurasia. To investigate the circulation of IDV among pigs in Italy, in the period between June 2015 and May 2016, biomolecular and virological tests were performed on 845 clinical samples collected from 448 pig farms affected by respiratory distress located in the Po Valley. Serological tests were conducted on 3698 swine sera, including archive sera collected in 2009, as well as samples collected in 2015 from the same region. Viral genome was detected in 21 (2.3%) samples from 9 herds (2%), while virus was successfully isolated from 3 samples. Genetic analysis highlighted that Italian swine IDVs are closely related to the D/swine/Oklahoma/1334/2011 cluster. Sera collected in 2015 showed a high prevalence of IDV antibody titers (11.7%), while archive sera from 2009 showed statistically significant lower positivity rates (0.6%). Our results indicate an increasing epidemiological relevance of the pathogen and the need for in-depth investigations towards understanding its pathogenesis, epidemiology and possible zoonotic potential of this emerging virus.

## Introduction

Influenza viruses are members of the *Orthomyxoviridae* family. They are enveloped, single stranded, negative sense RNA viruses with a segmented genome. Four genera of influenza virus are currently recognized: *Influenzavirus A* (IAV) and *Influenzavirus B* (IBV) that have eight gene segments, and *Influenzavirus C* (ICV) and *Influenzavirus D* (IDV)^1^ that have a genome consisting of seven segments. Moreover, ICV and IDV show only one surface glycoprotein, the hemagglutinin-esterase fusion (HEF) that exhibits receptor binding, receptor destroying and membrane fusion activities, thus combining the functions of HA and NA of influenza A and B viruses^2^. ICV is associated with mild infections of the upper respiratory tract in children^3^.

The primary host and reservoir of ICV are humans, but there is evidence suggesting it may also be able to infect other animal species. Specifically, serological studies have shown that antibodies against ICV are widely prevalent in pigs^4, 5^ and dogs^6, 7^. In 1981, ICV strains were isolated from pigs in Beijing and experimental studies demonstrated that they could be transmitted from pig-to-pig^8, 9^.

The first evidence of circulation of IDV dates back to 2011, when an influenza C-like virus was isolated from pigs exhibiting influenza-like symptoms in Oklahoma^10^ and soon after, from cattle in the USA^11, 12^, France^13^, China^14^, Italy^15^ and Japan^16^. Further investigations showed that this newly emerged virus was prevalent in samples from cattle diagnosed with bovine respiratory disease complex (BRDC)^12, 17, 18^. Thus, cattle were suggested to represent the main reservoir of the virus. Furthermore, the co-circulation of at least two distinct lineages of the virus was reported^12^. Genetic analyses showed that these new strains have about 50% amino acid homology with human ICV, while no cross-reactivity was observed to human ICV antisera^11^. Interestingly, this new influenza C-like virus exhibits a broader cell tropism compared to human ICV and is capable of infecting and transmitting by direct contact in ferrets, pigs^10^ and guinea pigs^19^.

In 2015, during routine diagnostic investigations of respiratory disease outbreaks in swine herds in Northern of Italy, the circulation of IDV was demonstrated both by molecular detection of viral genome and virus isolation^15^. Moreover, in the same period and region, IDV virus was detected in 27 (8.1%) out of 332 cattle herds investigated for respiratory pathogens^20^.

This study was conducted in the area of Northern Italy, the Po Valley, one of the most important swine-producing regions in Europe with more than 5 million pigs reared annually in approximately 7,200 herds, where IDV was isolated in 2015^15^. In order to better understand the epidemiology and importance of IDV in the Italian swine population, series of clinical specimens collected in 2015 and 2016 and sera collected in 2015, as well as archive samples dating back to 2009, 2013 and 2014 were examined in virological and serological investigations to determine the presence of IDV or IDV antibodies.

## Results

### Virological results

Between June 2015 and May 2016, 845 clinical specimens from pigs with respiratory disease symptoms from 448 farms were collected and tested for the presence of viral genome by Real Time RT-PCR. A total of 21 (2.3%) IDV-positive samples were found. These included, 14 out of 350 nasal swabs (4%), 3 out of 361 lung samples (0.8%) and 4 out of 134 (2.9%) oral fluid samples. The positive samples were traced back to 9 out of 448 herds (2%), identified from A to I (Table 1). The farms were located in Emilia Romagna (n.3), Lombardia (n.4) and Veneto (n.2). Viral isolation was obtained from 1 oral fluid and 2 nasal swabs from 3 herds. Accordingly, virus strains were named D/swine/Italy/199724-3/2015, D/swine/Italy/354017/2015 and D/swine/Italy/173287/2016. Virus isolation was obtained in Human Rectal Tumor (HRT-18G) cells both with and without trypsin treatment.

**Table 1:** Summary of investigations conducted in Real-Time RT-PCR IDV positive farms.

Herd	Farm/pigs type	IDV isolation	Clinical signs	Sample	IDV/PCR	APP	PCV2	PRRS	IAV
A	Fattening/weaned	No	none	oral fluids	+	nd	−	+	−
B	Farrow to finish/sow	D/swine/Italy/199724-3/2015	fever and abortion in sows	nasal swabs	+	nd	−	+	−
C	Farrow to finish/weaned	No D/swine/Italy/254578/2015*	respiratory disease	nasal swabs	+	nd	+	+	+
D	Fattening/growing pigs	D/swine/Italy/173287/2016	respiratory disease	nasal swabs and lungs	+	+	+	−	−
E	Fattening/weaned	No	respiratory disease	nasal swabs	+	nd	−	+	−
F	Fattening/weaned	No	mortality	lungs	+	−	−	−	−
G	Farrow to finish/weaned	No	acute respiratory disease	lungs	+	+	−	−	−
H	Farrow to finish/weaned	No	none	oral fluids	+	nd	−	+	−
I	Breeding gilts/gilts	D/swine/Italy/354017/2015	none	oral fluids	+	−	−	−	−

^*^Sequence obtained from clinical sample.

To better correlate respiratory disease with IDV infection, samples from IDV-positive herds, were also tested against other common swine respiratory diseases pathogens, while other epidemiological information were also incorporated in the investigation. The type and the location of these farms, as well as the disease reported and the investigations performed for the detection of other pathogens are summarized in Table 1.

An additional 54 clinical specimens from farms with respiratory disease collected in 2013 and 2014 were also screened by Real Time RT-PCR. No IDV viral genome was detected in any sample from this period.

### Serological results

**Figure 1:**
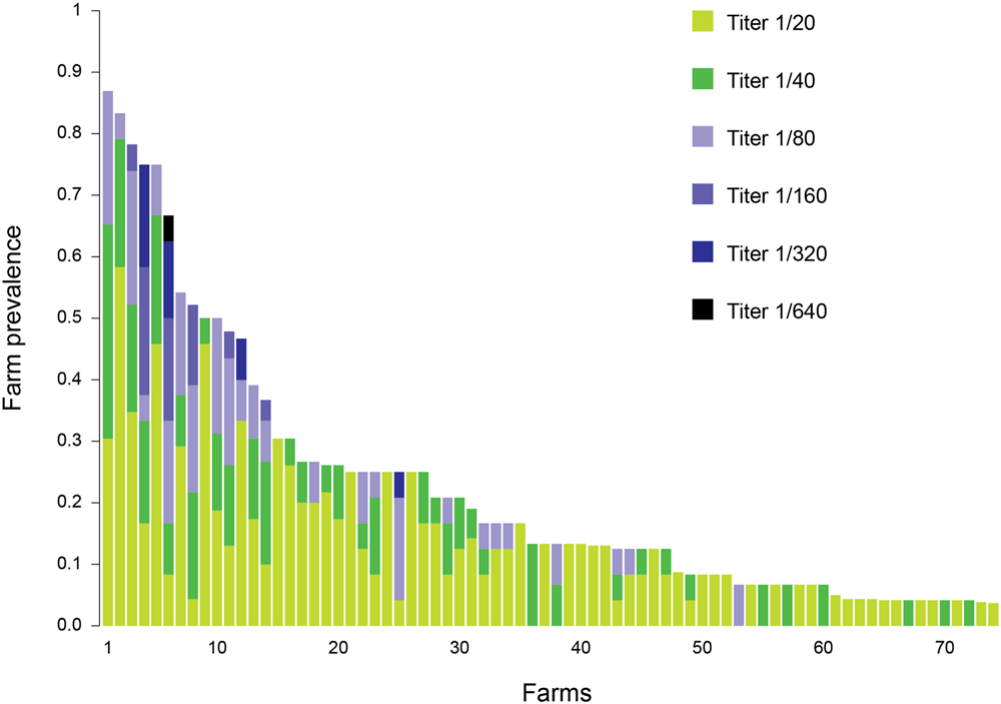
Serological results obtained performing HI test against IDV on 3115 sera collected in 74 swine herds (15–20 sera/herds) in 2015.

A total of 3106 swine sera collected at slaughter in 2015 from 143 herds were screened by hemagglutination inhibition (HI) test for antibodies against IDV. The HI test performed on the 3106 sera revealed the presence of antibodies against IDV in 364 samples (between-animal prevalence = 11.7% [CI95%: 10.6–12.9%]) from 74 herds (between-herd prevalence = 51.8% [CI95%: 43.6–59.8%]). As shown in Fig. 1, positive samples had HI antibody titers between 20 and 640. To exclude possible presence of non-specific inhibitors in low dilution of the tested sera, HI antibody specificity of titers  = 20 was confirmed by microneutralization assays.

Among the 74 sero-positive farms, 44 (59%) showed antibody titers ≥40, while 25 farms (34%) showed titers ≥80. Moreover, in 27 farms (36%), a serological prevalence higher than 25% of the tested animals was detected.

In addition, HI tests were performed on 90 sera collected from two herds (namely, farms B and C in Table 1) that were previously tested positive for IDV. In these herds 26 out of 90 sera were found sero-positive (prevalence = 28.9% [CI95%: 20.5–39.0%]). Among the positive samples, 15 sera had HI antibody titers of 20 (57%), 6 samples had titers of 40 (23%) and 5 showed titers of 80 (19%). The distribution of antibody titers in farms tested positive for IDV did not display significant difference with respect to the distribution of antibody titers in farms with positive sera from the serological screening (Wilcoxon rank sum test; W = 76324; p-value = 0.20).

Finally, HI test performed on 502 sera collected in 2009 from herds that had experienced respiratory symptoms identified only 3 samples (from 3 different herds) with low HI antibody titers (20 to 40) against IDV.

### Genetic analysis

**Figure 2:**
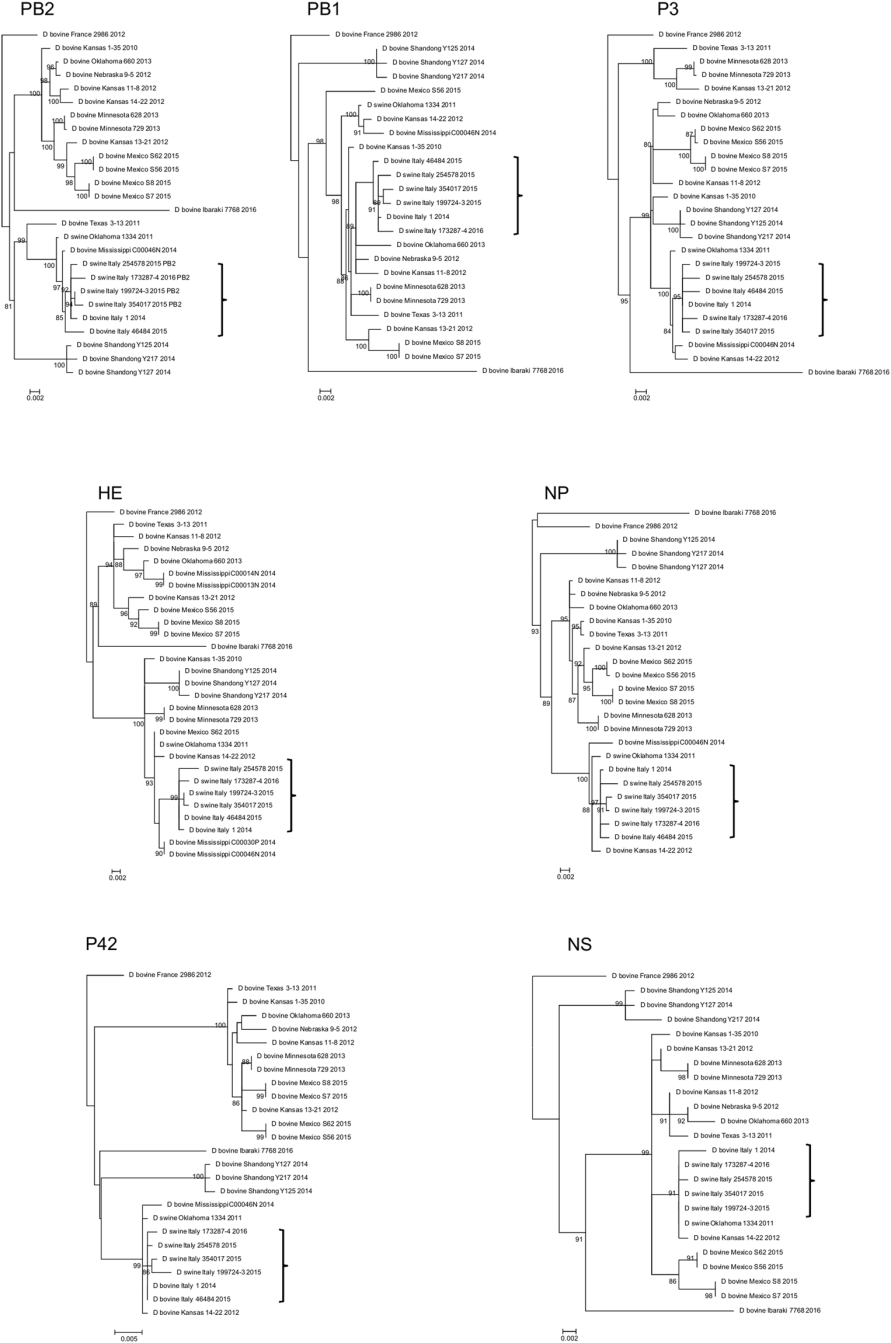
Phylogenetic trees of the seven IDV genes (PB2: 2318 nt, PB1: 2262 nt, P3: 2133 nt, HE: 817 nt, NP: 1659 nt, P42: 1164 nt, NS: 732 nt). Sequences are listed by their host, country, strain name and collection year. Scale bars indicate nucleotide substitutions per site. Influenza strains of this study are marked by a vertical brace.

Genetic analysis of all gene segments highlighted that Italian swine IDVs are very closely related to the D/swine/Oklahoma/1334/2011 cluster with no evidence of reassortment events. Phylogenetic relations are demonstrated in Fig. 2.

## Discussion

Although IDV was first isolated in swine, cattle were subsequently suggested as its main reservoir host and all ensuing studies have focused on this species. Some recent publications suggest that IDV has a global distribution in cattle, with high prevalence of sero-positive herds^11, 12^, while the virus is commonly detected in diagnostic submissions for bovine respiratory disease^12, 17, 18^. On the other hand, studies on the diffusion of this newly emerged pathogen in swine are generally missing both in Europe and North America.

Our results clearly demonstrate that IDV is currently circulating in swine herds in Northern Italy, in an area with high concentrations of both swine and cattle herds. However, it is important to notice that the two species are not mixed by farming practices, but one cannot exclude virus mechanical or airborne transportation. Passive surveillance revealed virus presence in 9 of the 448 pig farms investigated (2%). Furthermore, parallel investigation of swine and bovine archival samples should be conducted to fully clarify when the virus was introduced in cattle and whether its presence in swine might be correlated with periodical outbreaks in cattle.

Although similar quantities of low (lungs) and upper (nasal swabs) respiratory tract samples (361 to 350) were examined, the majority of IDV-positive samples were nasal swabs (14 against 3). This finding is in agreement with the results of experimental infections of pigs with IDV conducted by Hause and coworkers^10^, in which virus was only detected in the upper respiratory tract. This confirms that IDV replication occurs mainly in the upper respiratory tract and indicates that nasal swabs are the preferred sample for diagnosis. On the other hand, it is important to underline IDV was also detected in three lung samples, suggesting that infection can also reach the lower respiratory tract either by active virus replication, or possibly passively by muco-ciliary transportation.

The presence and circulation of IDV in Italian pig farms is further highlighted by the serological results presented in this study. Specifically, 36.5% (27/74) of the positive farms examined in 2015, showed a positive rate >25% of the tested sera (Fig. 1). Among these, 17 (63%) showed sera with titer ≥80. Overall, 30 of the 74 positive farms showed sera with positive titers not higher than 20, which is in agreement with the findings of Hause and coworkers^10, 11^. This low seroconversion was confirmed by the HI test results in farms known to be infected by IDV, with 57% of the positive sera showing a titer of 20.

Serological data on samples collected from 2009 suggest that IDV emerged in the region recently, while its prevalence has been steadily increasing over the past three to four years. In fact, only 3 of the 502 sera from 2009 showed detectable titers to IDV (prevalence = 0.6% CI95%: 0.2–1.7%). This significant increase in virus circulation is also confirmed by the results of the retrospective virological study that examined samples collected in 2013 and 2014, and in which all 54 samples examined were negative.

The etiological role of the IDV in respiratory distress in the species is an important question posed by the frequent detection of virus and IDV-specific antibodies in swine herds where respiratory is reported. In this study, co-infections with other swine respiratory pathogens were identified in eight out of nine herds IDV-positive herds, while in one farm, otherwise healthy gilts were found positive for IDV alone (Table 1). To offer a definite answer to this question, more pathogenesis and transmission experiments in pigs must be conducted, including infections with IDV alone or combined with other pathogens to investigate possible synergy. While IDV is a novel virus, its importance and potential for interspecies transmission should not be underestimated.

The circulation of at least two distinct IDV lineages has been identified in the U.S. cattle population^12^. Phylogenetic analysis revealed that Italian IDV strains isolated in this study and previous investigations^15^ belong to D/swine/Oklahoma/1334/2011 genetic cluster. However, the possible introduction of a second IDV lineage in the country, the endemic status of IDV in cattle and, to a lower degree, in pigs, together with the proven capacity of the virus to reassort^12, 14^, underline the need to continuously monitor susceptible species and to track virus evolution.

Additionally, the virus seems able to replicate in other animals, including small ruminants^21^, ferrets^10^, guinea pigs^19^ and possibly humans. A study by Hause *et al*.^10^ showed a low percentage (1.3%) of positivity in a set of 316 human sera, while a more recent survey in Scotland was unsuccessful in identifying IDV from archived human respiratory samples^22^. However, the sero-prevalence of IDV among cattle workers in Florida was found to be as high as 91%^23^. Therefore, the zoonotic aspect of this emerging pathogen cannot be excluded or neglected and further investigations are required, especially in people with occupational frequent exposure to cattle and swine.

## Materials and Methods

### Sample Collection

#### Clinical samples

Between June 2015 and May 2016, a total of 845 samples from 448 pig farms located in the Po Valley area were collected. The area is comprised of the regions of Piemonte, Lombardia, Emilia Romagna and Veneto. Furthermore, the majority of cattle in Italy are also raised in these regions. Samples were collected by field veterinarians as part of the routine diagnostic protocol for the control of respiratory diseases in swine. Specifically, the clinical specimens included nasal swabs (n.350), oral fluids (n.134) and lung samples (n.361). From the laboratory’s archive, an additional 54 nasal swabs collected from the same region in 2013 (n.32) and 2014 (n.22) respectively, were also selected for virological testing.

#### Serum samples

In the serological screening, 3106 samples coming from 143 herds in Northern Italy (representing ~5% of the 2900 finishing farms in the same region), were randomly collected at slaughter as part of a routine screening program for Aujeszky virus infection, between June and December 2015. The median number of samples examined in each herd was 24 (range 10–30). By assuming 9.5% as the expected seroprevalence in pigs as indicated by Hause *et al*.^10^, our study design provides a desired precision equal to 1% in the estimate of between-animal seroprevalence and (considering 24 sera/herd) a probability higher than 90% to find positive sera in a herd with seropositive animals.

In addition, to estimate the distribution of antibody titers in farms positive to IDV, we collected 90 samples in 2015 from two farms (namely, B and C Table 1), just after IDV had been there detected.

Confidence intervals in the observed prevalence were estimated by using binomial approximation. Differences in the empirical distributions of antibody titers between farms tested positive for IDV and farms with positive sera from the routine screening through were identified with the Wilcoxon rank sum test. Statistical analyses were performed in the R 3.2.0 environment.

Finally, to evaluate whether IDV was previously circulating in Northern Italy, we analyzed 502 samples collected in 2009 from 25 herds (range: 15–25 samples/herd) where respiratory diseases occurred.

#### Real-Time RT-PCR

Viral RNA was extracted from clinical samples using One-For-All Vet Kit (Qiagen, Hilden, Germany) according to the manufacturer’s instructions. The subsequent Real-Time PCR was performed as previously described by our group^24^.

#### Virus isolation

Samples positive by Real-Time RT-PCR were tested for virus isolation in Human Rectal Tumor cells (HRT-18G) (ATCC, Manassas, VA) as described by Ferguson *et al*.^25^. To maximize chances of virus isolation, samples were tested both with and without trypsin added to the culture medium. Following infection, incubation was prolonged up to 5 days, in the absence of cytopathic effect. Two serial passages were performed for each sample. Confirmation of viral replication was performed using hemagglutination test and Real-Time RT-PCR test at each passage.

#### Other diagnostic tests

In order to correlate IDV with respiratory disease and to exclude other causative agents, clinical specimens from IDV positive herds were also subjected to standard bacteriological and/or virological PCR tests against the most common swine respiratory pathogens. These included: *Actinobacillus pleuropneumoniae* (APP)^26^, swine IAV^27^, Porcine respiratory reproductive (PRRS) (LSI VetMAX™ PRRSV EU/NA Real-Time PCR Kit, Thermo Fisher Scientific, Waltham, MA USA) virus and Porcine Circovirus 2 virus (PCV-2)^28^.

#### Hemagglutination and hemagglutination inhibition tests

Hemagglutination (HA) and hemagglutination inhibition (HI) tests were performed as described in standard protocols^29^. HI assay was performed using 0.5% turkey red blood cells in U-bottom 96 well plates. Briefly, sera were treated 1:5 with receptor-destroying enzyme (RDE) (Sigma-Aldrich, Milan, Italy) at 37 °C overnight, followed by heat inactivation at 56 °C for 30 min. After treatment with 50% turkey red blood cells, the sera were diluted to a final concentration of 1:10 with sodium citrate. The assay was conducted at room temperature starting from dilution 1:20 to 1:640 for detection of D/swine/Italy199724–3/2015 specific antibodies. Samples showing antibody value ≥ 20 were considered positive. HI titers were expressed as the reciprocal of the highest dilution of serum that completely inhibited hemagglutination (4 HA units were used). A negative serum, as well as a swine polyclonal antiserum generated using D/swine/Italy199724-3/2015 were used as controls in the HI assay. The antiserum was produced in IAV and IDV negative pigs by intra-tracheal inoculation of live virus (2 ml 10^−5,5^TCID_50_) and boosting by intramuscular injection (2 ml with Freund’s complete adjuvant) two weeks later. The polyclonal positive control antiserum was tested against swine IAVs circulating in Italy (A/swine/Italy/257605/2010 H1N1, A/swine/Italy/284922/2009 H1N2, A/swine/Italy 312583/2009 H3N2) showing no cross-reactivity. Serological cross reactivity against ICV has not been considered because circulation of ICV in swine in Italy has not been demonstrated. Moreover Hause *et al*.^11^ showed no cross reactivity between IDV and ICV in human sera.

#### Microneutralization test

A microneutralization assay was performed as described for Influenza A virus^30^. Virus stock was titrated with HRT-18G cells using a lysis by boiling of the cell lysate^31^ followed by the Real-Time RT-PCR assay^24^ to identify infected and non-infected culture wells. The TCID_50_ was calculated by the method of Reed-Muench^32^. Infection was performed as described for Influenza A virus^33^. An inoculum containing 1000 TCID_50_/50 µl of IDV was mixed in duplicate, in a 96-well culture plate, with a two-fold dilution series (from 1/10 to1/160) of 15 sera showing HI titer 1/20. Incubation was performed for 1 h at 37 °C and then 100 µl of HRT-18G cells (1.5 × 10^4^ per well) were added. After incubation of 72 h at 37 °C viral RNA was extracted from cell culture supernatant diluted 1:1 v/v in water and boiled 10 min at 95 °C as previously described^31^. Viral infectivity was assessed by RT-PCR^24^ using 5 µl of boiled sample. Sera dilution with at least 90% inhibition of the RT-PCR signal were considered as positive.

### Ethics statement

Animal experiments were conducted at IZSLER, Brescia, in compliance with the Ethical Committee for Animal Experimentation of the Institution (Istituto Zooprofilattico Sperimentale della Lombardia ed Emilia Romagna). The treatment, housing and husbandry conditions conformed to the National Ministry of Health Guidelines. Animal care and procedures were under the supervision of the Ethical and Animal Welfare committee of the IZSLER (number approval: 12–3–13).

### Genetic analyses

Viral RNA was extracted from cell cultures or clinical samples using the One-For-All Vet Kit (Qiagen, Hilden, Germany) according to the manufacturer’s instructions. RT-PCR of all seven genome segments was performed as previosly described 10 using SuperScript® III One-Step RT-PCR System with Platinum® Taq High Fidelity (Thermo Fisher Scientific, Waltham, USA). RT-PCR products were purified with NucleoSpin® Gel and PCR Clean-up (Macherey-Nagel, Düren, Germany). DNA libraries were made with NEXTERA-XT kit (Illumina Inc. San Diego, CA, USA) according to manufacturer’s instructions. Libraries were sequenced on a MiSeq Instrument (Illumina Inc. San Diego, CA, USA) using a Miseq Reagent Kit v2 in a 250 cycle paired-end run. Data were *de-novo* assembled and analyzed by the NextGen Lasergene application (DNASTAR, Madison, USA) in the Lasergene Package software (Ver 12.0). Gene sequences from the Italian IDVs and reference IDV sequences retrieved from Genbank were aligned with ClustalW using MEGA5^34^. Phylogenetic trees of the individual segments were inferred with the maximum likelihood (ML) method implemented in IQ-TREE package 0.9.6 24. The robustness of the ML trees was statically evaluated by bootstrap analysis with 1000 bootstrap samples.

### Data availability

Sequences for IDVs of this study were deposited in GenBank under accession numbers KX768817-KX768844, KT592530-KT592536.
